# Effects and Effectiveness of Two RNAi Constructs for Resistance to *Pepper golden mosaic virus* in *Nicotiana benthamiana* Plants

**DOI:** 10.3390/v5122931

**Published:** 2013-11-28

**Authors:** Diana Medina-Hernández, Rafael Francisco Rivera-Bustamante, Francisco Tenllado, Ramón Jaime Holguín-Peña

**Affiliations:** 1 Laboratorio de Fitopatología, Centro de Investigaciones Biológicas del Noroeste, Instituto Politécnico Nacional 195, Col. Playa Palo de Santa Rita, La Paz, Baja California Sur, 23096, Mexico; E-Mails: jholguin04@cibnor.mx (R.J.H.P.); dmedina@cibnor.mx (D.M.H.); 2 Departamento de Ingeniería Genética, Centro de Investigación y de Estudios Avanzados del IPN, Unidad Irapuato, Km. 9.6 Libramiento Norte, Irapuato, Guanajuato, 36821, Mexico; E-Mail: rrivera@ira.cinvestav.mx (R.F.R.B.); 3 Departamento de Biología Medioambiental, Centro de Investigaciones Biológicas, Ramiro de Maeztu 9, Madrid, 28040, Spain; E-Mail: tenllado@cib.csic.es (F.T.)

**Keywords:** Hairpin RNA, Mixed infections, PepGMV, RNAi constructs, ToChLPV, Viral load

## Abstract

ToChLPV and PepGMV are *Begomoviruses* that have adapted to a wide host range and are able to cause major diseases in agronomic crops. We analyzed the efficacy of induced resistance to PepGMV in *Nicotiana benthamiana* plants with two constructs: one construct with homologous sequences derived from PepGMV, and the other construct with heterologous sequences derived from ToChLPV. Plants protected with the heterologous construct showed an efficacy to decrease the severity of symptoms of 45%, while plants protected with the homologous construct showed an efficacy of 80%. Plants protected with the heterologous construct showed a reduction of incidence of 42.86%, while the reduction of incidence in plants protected with the homologous construct was 57.15%. The efficacy to decrease viral load was 95.6% in plants protected with the heterologous construct, and 99.56% in plants protected with the homologous construct. We found, in both constructs, up-regulated key components of the RNAi pathway. This demonstrates that the efficacy of the constructs was due to the activation of the gene silencing mechanism, and is reflected in the decrease of viral genome copies, as well as in recovery phenotype. We present evidence that both constructs are functional and can efficiently induce transient resistance against PepGMV infections. This observation guarantees a further exploration as a strategy to control complex *Begomovirus* diseases in the field.

## 1. Introduction

*Begomovirus* is the largest genus in the *Geminiviridae* family and includes approximately 192 species that infect dicot plants; are transmitted by the *Bemisia tabaci* whitefly species complex, and can be either monopartite (containing a single DNA-A component) or bipartite (DNA-A and DNA-B) [1]. In the last two decades, *Begomoviruses* have become one of the most important groups of plant viruses in tropical and subtropical regions and are responsible for the emergence of new diseases that cause important yield losses [2,3]. *Pepper golden mosaic virus* (PepGMV) is one of the most widely distributed *Begomoviruses* species affecting tomato (*Solanum lycopersicum* L.), and pepper (*Capsicum annuum* L.) and it is disseminated over a large geographic area spanning southwestern United States, Mexico and Central America [4]. PepGMV, has been found co-infecting tomato with the bipartite *Begomoviruses* *Pepper huasteco yellow vein virus* (PHYVV) [5,6] and *Tomato yellow leaf curl virus* (TYLCV) [7]. These co-infections appear as the most devastating viral disease in Mexico with important epidemiological implications since the disease spreads faster and causes more severe symptoms than those caused by single infections. Mixed viral infections have been increasingly found in a wide range of hosts, including pepper, tomato, green tomato, “tomatillo” (*Physalis ixocarpa* Brot.) and tobacco [4,5]. Another virus, the *Tomato chino La Paz virus* (ToChLPV), has been found infecting tomato plants in the Baja California Peninsula [8]. In addition, ToChLPV was found in co-infection with TYLCV in tomato and tomatillo, Mexican thistle (*Solanum rostratum* Dunal), and pepper plants in Sinaloa, San Luis Potosi, and Baja California Sur, respectively [9,10,11]. The presence of these interacting viruses represent a risk for the emergence of new and complex diseases, and have important implications in the sustainable control systems of the disease [3,12,13]. 

A biotechnological tool that has been used to induce pathogen-derived resistance against begomoviral diseases in plants is the RNA interference (RNAi) [14,15,16]. RNAi is a very adaptable strategy because its specificity is dictated by the sequence of the viral genome itself, and the small interference RNA (siRNA) that serves as a guide and can down-regulate gene expression and viral DNA accumulation [17]. A variety of geminivirus resistance strategies have been tested such as the expression of mutated viral proteins [18], antisense RNAi silencing transcript of viral sequences [15,19], 21–25 nt-long siRNAs [20], artificial micro-RNAs (amiRNAs) [21], and chimeric transgene constructs [22]. Commonly, these RNAi strategies have been used successfully with the entire, partial or mutated sequences of AC1 (Rep) or AV1 (CP) genes and the non-coding intergenic region (IR) [23,24,25]. The AV1 gene was the first gene to confer resistance against homologous viral infections (closely related species) and remains one of the most widely used. In some cases protection is broad and effective against several strains of the virus from which the AV1 gene is derived [23]. However, this strategy has not resulted in a significant level of resistance for bipartite *Begomoviruses*. The AC1 gene has been an excellent target to generate broad resistance because it: i) is highly conserved; ii) is involved in the modulation of geminiviral gene expression, and iii) would impact viral replication by reducing viral DNA accumulation [26,27,28,29].

Effects and effectiveness to evaluate transgenes are commonly based on severity of symptoms and incidence of the disease, which are analyzed by PCR and dot blot hybridization [30]. In addition to results based only on visual assessments and qualitative or semi-quantitative data generating only subjective results, interactions of other intrinsic features such as recognition of specific targets, symptoms recovery and viral DNA accumulation are difficult to unravel [31]. Real-time PCR (qPCR) offers greater sensitivity to determine the viral DNA accumulation and is an important tool to establish the relation with the resistance [32]. Resistant plants are those that can suppress the multiplication of a virus and hence suppress the development of disease symptoms. Tolerance is when plants show no symptoms, but the viral DNA is present [33]. Thus, qPCR analysis can be a promising approach to study the efficacy of heterologous constructs for virus resistance, epidemiological studies and to establish antiviral therapies. The responses of plants to viral infections and to pathogens in general, have been studied extensively using several tools (e.g. microarrays, RNA-seq) that allow the evaluation of the transcriptome expression in these interactions [34]. These tools are also ideal in examining the presence of RNA silencing genes in a host and their expression during virus infections [35]. 

The present study reports the engineering of two RNAi constructs based on the AC1-IR-AV1 region of PepGMV and ToChLPV. We evaluated their efficacy to induce resistance to PepGMV infections in *Nicotiana benthamiana* plants by *Agrobacterium*-mediated transient assay. Also, we analyzed by microarray hybridization the altered mRNAs to detect key components related to silencing pathways.

## 2. Results

### 2.1. Development of CIRP (Construct of the Intergenic Region of PepGMV) and CIRT (Construct of the Intergenic Region of ToChLPV) Constructs

To evaluate the resistance efficacy against *Pepper golden mosaic virus* (PepGMV), we engineered two hairpin-type (hpRNA) constructs designed to express dsRNA of the AC-IR-AVI segment of PepGMV and ToChLPV. The constructs were designed to express an intron-hairpin comprising the ≈146 nts of the 5´ end of the AV1 gene, the entire (326 nts) intergenic region (IR) and 714 nts of the 5´ end of the AC1 gene, the AC1-IR-AV1 segment total comprising 1186 nts (Figure S1). The presence of transgenes was detected by PCR. 

### 2.2. Resistance Assays and Determination of Severity, Incidence and Viral Load

**Figure 1:**
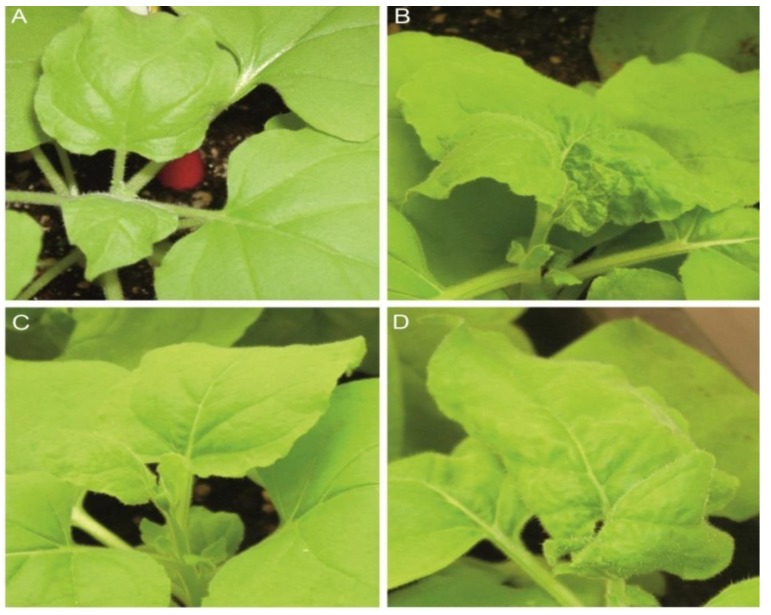
Comparison of the symptoms of PepGMV-inoculated *N. benthamiana* plants in different bioassay groups. A) Healthy plant used as negative control. B) Plant 10 days after inoculation with PepGMV infectious clone (positive control) showing severe leaf curling and distortion of the systemic, young leaves. C) Plant agro-infiltrated with CIRP construct challenged with PepGMV showing no symptoms. D) PepGMV inoculated, CIRT-protected plant showing light to mild symptoms

The resistance generated by CIRP (Construct of the Intergenic Region of PepGMV) and CIRT (Construct of the Intergenic Region of ToChLPV) was determined by challenging *N. benthamiana* plants with an infectious clone derived from PepGMV. In the resistance analysis, all the non-infected plants (negative control) showed a normal phenotype (Figure 1A). All positive control plants (inoculated with only PepGMV) developed typical leaf curl symptoms in emergent leaves within 10 dpi (Figure 1B, Table S1).

Most *N. benthamiana* plants infiltrated with either CIRP or CIRT and inoculated with PepGMV, did not show severe symptoms or showed only attenuated symptoms (Figure 1C, 1D and Table S1). We observed that 5 out of 8 CIRP-protected plants remained symptomless over a period of 10 days, and two plants exhibited delayed or attenuated symptoms (foliar severity range 0.5, Table 1) compared with PepGMV-inoculated plants (2.5). With CIRT plants, 3 out of the 8 plants remained symptomless and three showed mild symptoms and foliar severity range of 1.37. The decrease in severity symptoms (efficacy) was 80% in plants protected with CIRP and 45% in plants protected with CIRT (Table 1). According to the Mann–Whitney (P≤0.05) test, we found differences in severity between plants protected with CIRP and the positive control (unprotected, infected plants). However, no significant differences were observed between plants protected with CIRT and the positive control. The presence of PepGMV was confirmed by PCR using degenerated primers [36], with a 550 bp amplicon only detected in symptomatic plants. Also, the absence of PepGMV was confirmed in all the symptomless plants by PCR amplification (Table S1). In incidence reduction, we found a significantly higher efficacy of 57.2 % in plants protected with CIRP compared to 42.6% in plants protected with CIRT (Table 1). 

For viral load, the results of the interpolation between the logarithm (log) of the amount of starting quantities (SQ) to the amount of the endogenous 18S rRNA gene was y = –3.450x + 13.0, with R² = 0.999 (efficiency of 94.9%). The standard curve to PepGMV AV1 gene was y = –3.385x + 18.5, with R² = 0.989 (efficiency of 97.4%).

**Figure 2:**
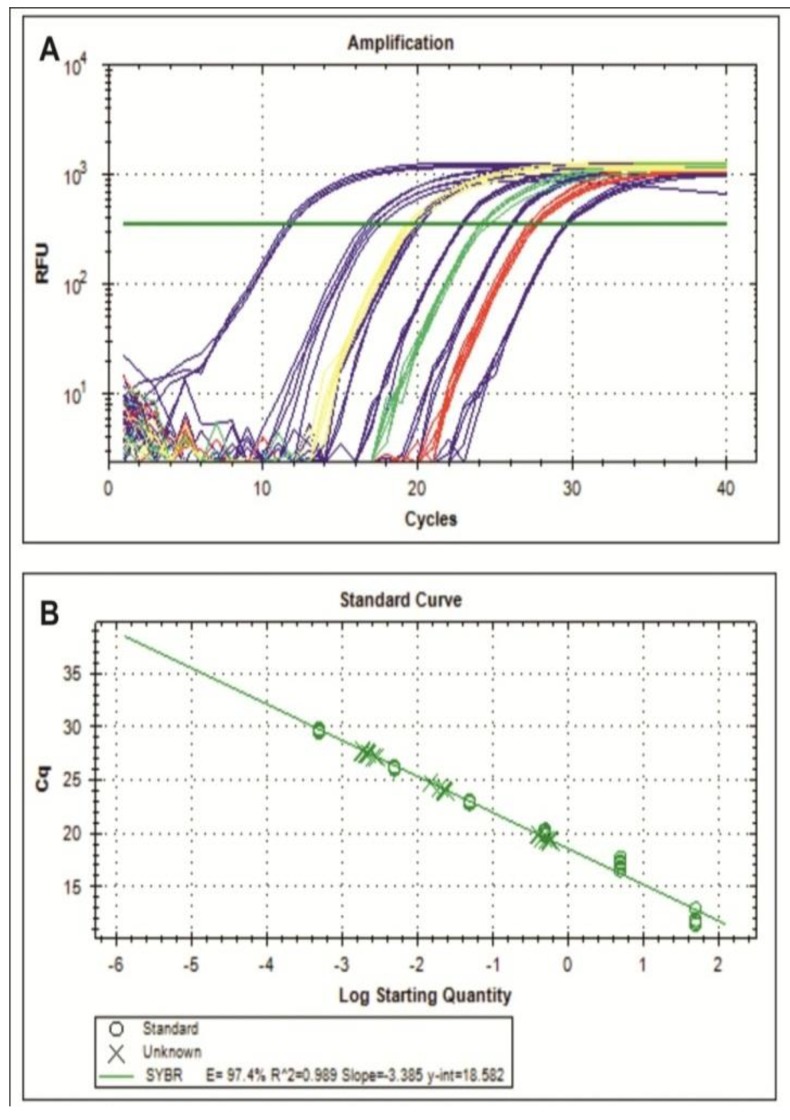
Real-time PCR amplification. A) Typical amplification plot of qPCR to determine mean threshold cycle (C*_t_*) values. Blue line: serial dilutions of PepGMV AV1 gene plasmid DNA. Yellow line: positive control plants. Green line: plants protected with construct CIRT. Red line: plants protected with construct CIRP. B) Standard curves were obtained by linear regression of the Ct values of the five standard-dilution replicates over the log SQ of the copies of DNA targets.

The curves showed high correlation coefficients and were linear over the concentration range with standard and examined DNA. The melting curves of the samples and amplified serial dilutions of the gene AV1 showed that the primers did not generate nonspecific products during the amplification. In both protected groups of plants, the results suggest that titers of PepGMV are at very low levels, compared to positive control (Table 1, Figure 2A). The result of viral DNA accumulation was positively correlated with symptom severity, with the lower values found in symptomless plants or in plants that exhibited mild symptoms (Table S1). We observed high efficacy in plants protected with both constructs in decreasing the viral load. The efficacy observed with CIRT was 95.6% and 99.56% with CIRP (Table 1). Also, we found a co-relationship with the significant reduction in the severity (45 to 80%) and incidence (42.6 to 57.2%) compared with control plants. The average of absolute quantification of viral DNA was different (P < 0.05 ANOVA test) in all treatments. The highest value observed was in the positive control with 5.22 × 10^-1^ copies. With CIRT we observed 2.27 × 10^-2^ copies and 2.20 × 10^-3^ copies in CIRP (Figure 2B). Thus, viral accumulation was reduced 19.3- and 237.2-fold in CIRT and CIRP, respectively, in terms of the control plants. The viral copies with CIRP were reduced 12.3-fold compared with CIRT.

**Table 1:** Summary of findings on comparative effectiveness of two RNAi constructs to reduce symptoms, incidence and viral DNA accumulation in experimental PepGMV infections.

		Severity				Incidence				Viral load		
Constructs		Foliar severity range ^1^	Percentage of foliar damage (%) ^2^	Efficacy (%) ^3^		PCR (+/-)	Adjusted data (%)	Efficacy (%)		Copies	Folds	Efficacy (%)
CIRT		1.375	55 ^a^	45		4/4	57.4 ^c^	42.6		2.27 × 10^-2 c^	19.3	95.6
CIRP		0.50	20^ b^	80		3/5	42.8 ^b^	57.2		2.20 × 10^-3 b^	237.3	99.56
Positive control		2.5	100^ a^	0		8/0	100 ^a^	0		5.22 × 10^-1 a^	1	0

^1^ Data represent the mean of replicates. ^2^ Data were converted to percentages with the formula present in methodologies and adjusted data as maximum value according to positive control (non-protected plants that were inoculated with infectious clone PepGMV). Lowercase letters indicated the significant difference among the constructs and positive control at P ≤ 0.05 (Mann–Whitney test).

### 2.3. Gene Expression Related to RNAi

To determine whether the efficacy of CIRT and CIRP was due to RNAi, we analyzed the transcriptome of *N. benthamiana* by microarray hybridization to detect the presence of up-regulated key components of the silencing pathways. Microarray analysis determined the presence and expression of some of the key components that activate the RNAi mechanism. These components were found on the lower center (z-score 0.5–1). Components common to both CIRT and CIRP treatments were: RNA polymerase IV, DCL3, AGO4, DRD1, DDM1, CMT3, and MET1. Other components of RNA silencing were differentially expressed (greater 1.5 up) in either CIRT or CIRP treatments. Components particular to CIRT included AT2G35160.1, which encodes SU(var)3-9 homolog 5 (SUVH5); AT3G15390.1, encoding silencing defective 5 (SDE5); AT3G49500.1, encoding RNA dependent RNA polymerase (RDR6); AT4G14200.1, encoding pentatricopeptide repeat (PPR), and AT5G23570.1, which encodes SGS3. Components particular to CIRP included AT1G08060.1, encoding Morpheus molecule maintenance of methylation (MOM, MOM1); AT1G09700.1, encoding dsRNA-binding protein 1 (DRB1); AT3G22680.1, encoding RNA-directed DNA methylation 1 (RDM1); AT3G42670.1, encoding chromatin-remodeling 38 (CHR38), and AT4G11130.1, encoding RNA-dependent RNA polymerase 2 (RDR2).

### 2.4. Discussion

Our results provide evidence that both CIRT and CIRP constructs are highly efficient to suppress the multiplication of a viral genome and can be explored in the control against PepGMV infections and other related species involved in mixed infections. Our results agree with several study groups [37], indicating that homologous recognition is highly efficient to induce RNAi. According to the hypothesis, we obtained high efficacy of 99.56% (Table 1) in reducing viral DNA with homologous construct CIRP. Indeed, complete immunity was observed in most of the protected plants, and viral accumulation was reduced 12.3-fold compared with CIRT and 237.3-fold compared with CIRT and positive control plants, respectively. With the heterologous CIRT construct we obtained an efficacy of 95.6% and viral DNA accumulation was reduced 19.3-fold compared with the positive control.

**Figure 3:**
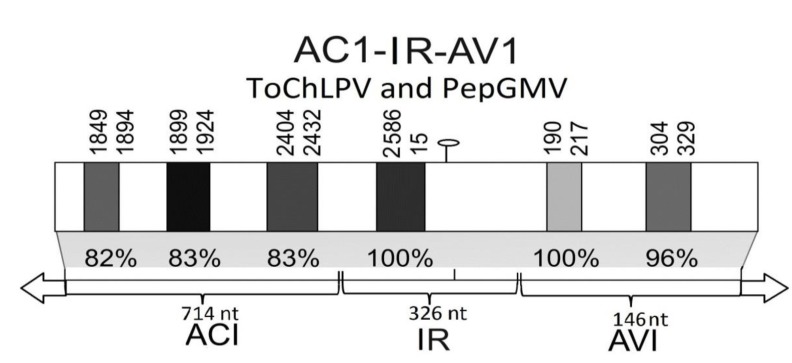
Alignment of region AC1-CR-AV1 from ToChLPV and PepGMV**.** The six regions highlighted in the gray boxes indicate fragments longer than 22 nts of both viruses, which show homology from 82% to 100%.

Previous studies mentioned that to obtain optimal results with heterologous RNAi constructs, the nucleotide (nt) identity should be at least 85% [38]. Nucleotide alignments of AC1-IR-AV1 segments of PepGMV and ToChLPV show identity of 89%. Moreover, we found two 22 nt-long stretches with 100% similarity: one with 96% and three with 82% (Figure 3). According to the observed, the homology was sufficient to trigger an effective reduction in the expression levels of the disease and to generate heterologous recognition between CIRT and PepGMV, activating the RNAi mechanism, to trigger an effective reduction of the disease in CIRT-protected plants. These results, together with those published by others [26,27,28,29,39], add to the conclusion that strong cross-protection can be induced by transgenes with sequences AC1, IR or AV1, likely producing high population of siRNAs that efficiently activate the silencing process.

In a partial region of the AC1 gene, we found three ≈24 nt-long (Figure 3) that have 82% homologous in ToChLPV and PepGMV. The success in the use of the AC1 gene is that it specifically targets the replicase gene with the consequent impact in viral DNA replication [27]. On the other hand, other studies [24,25] have shown that AV1-type constructs can induce the silencing response due to the specific recognition of the AV1 gene. Indeed, we found two ≈24 nt-long sequences in the first 146 nts that were 100% homologous in ToChLPV and PepGMV (Figure 3).

It has been documented that resistance is broad and effective against strains of the virus from which the AV1 gene is derived, or even against closely related virus species. The use of the non-coding IR region is an interesting strategy to induce broad resistance against other heterologous geminiviruses. This is because the generation of 24 nt siRNAs derived from activity of DCL3 modifies methylation processes, which is considered the principal mechanism of antiviral defenses through transcription gene silencing [16].

In addition, it has been reported that dsRNA can be generated by single and specific siRNA that can be elongated and consequently generate more specific siRNAs, thereby imparting resistance not only against homologous but also against heterologous virus species [26]. The analysis of the transcriptome in CIRP- and CIRT-protected plants revealed the expression of some key components of the RNAi pathway involved in the formation of primary and secondary viral siRNAs. These RNAi components cause changes in the conformation of local chromatin and on the epigenetic regulation resulting in gene silencing [40]. This provides evidence of protection to PepGMV based on RNAi in CIRP- and CIRT-treated plants. Moreover, we observed that the up-regulated components in CIRT-protected plants were different from those observed in CIRP-protected ones. This suggests that the activation of the response also depends on the specificity of the sequence used to generate the dsRNA. Disease symptoms can be the result of specific interactions between virus and host components [41]. The CIRT construct showed a relatively low efficacy in reducing symptom severity and incidence, but a high efficacy at reducing the viral DNA. This can be explained because severity is calculated with qualitative values and therefore is somehow subjective. Also, the percentages of infection based only in conventional PCR depend on the intensity of the signal, and results often lead to underestimate quantitative differences [42]. qPCR is a valuable tool in comparing homologous/heterologous RNAi constructs since it cannot only quantify viral DNA accumulation, but also correlate, more precisely, viral load and the severity of symptoms [43]. In summation, we demonstrated that heterologous RNAi-type constructs are highly efficient to induce cross-protection and can be an important tool in the strategy for control of PepGMV and other *Begomovirus*-complex diseases.

## 3. Experimental Section

### 3.1. Development of RNAi Constructs

The construct designed with PepGMV is homologous in respect to the challenging virus (PepGMV) and it was named CIRP (Construct of the Intergenic Region of PepGMV). The second construct derived from ToChLPV is considered heterologous and it was named CIRT (Construct of the Intergenic Region of ToChLPV). The generated amplicons to be used in the constructs were based on the degenerate primers PAL1v1978-PAR1c496 [44] with added attB sequences. The amplification was carried out with Phusion High-Fidelity DNA Polymerase (Finnzymes) with the following PCR parameters: 1 cycle of 98° C/30 s, followed by 30 cycles of 98 °C/10 s, 60 °C/30 s, and 72 °C/30 s. The generated products were introduced by BP recombination into the pDONR™ 207 using the Gateway system (Invitrogen) according to the manufacturer’s instructions. The resultant products were recombined subsequently into binary vector pH7GWIWG2(II) [45], using LR Clonase™ enzyme (Gateway®), generating the two expression vectors: CIRP and CIRT. CIRP and CIRT contained inverted repeats sequences of the AC1-IR-AV1 region of each virus, including an intron as a spacer Restriction digestion analysis was performed with *Xba*l to confirm the presence of the sense and antisense arms and orientation of the inverted repeat sequences. CIRP and CIRT were transformed into *Agrobacterium tumefaciens* GV2260 by electroporation (Bio-Rad Gene Pulser II Electroporator). 

### 3.2. Design of the Experimental Bioassay

#### 3.2.1. Inoculation Assays

*N. benthamiana* seeds were grown for 30 days in chambers at 28 °C with a photoperiod of 16 h light: 8 h dark. To evaluate the protective effect against PepGMV infection and the effectiveness in the activation of the RNAi response, we conducted a completely randomized study consisting of four treatments: (1) CIRP, plants agro-infiltrated with CIRP and then inoculated with PepGMV four days after infiltration; (2) CIRT, plants agro-infiltrated with CIRT and then inoculated with PepGMV four days after infiltration; (3) positive control, plants agro-infiltrated with the empty vector (pH7GWIWG2(II)) and then inoculated with PepGMV four days after infiltration; and (4) negative control, plants agro-infiltrated with the empty vector but not inoculated with PepGMV. Eight plants per treatment were agro-infiltrated in the four-leaf stage according to a previously published protocol [46]. The inoculations of infectious clones PepGMVAdimpBS and PepGMVBdimpBS (each with two genomes in tandem of PepGMV DNA A and DNA B, respectively) were performed by biolistic inoculation on the third and fourth agro-infiltrated leaves according to the procedure described in [47]. The data were recorded at 10 days post inoculation (dpi). 

#### 3.2.2. Evaluation of variables

##### A. Severity

Symptom severity was evaluated based on the following adjusted scale: 0, no visible symptoms; 1, presence of visible mild symptoms; 2, some yellowing and minor curling of leaf ends; and 3, severe symptoms of curling throughout the plant [5]. The percentage of severity was calculated according to [48] with the formula S = (Σi/N [VM])*100, where S = percentage of severity, Σi = sum of observed values, N = number of plants observed, and VM = full-scale value. To determine the significant differences between groups, we performed the non-parametric Mann–Whitney test (P ≤ 0.05).

##### B. Incidence

The incidence was calculated based on the detection of viral transcripts by PCR using the degenerate primer pair AC1048-AV494 [36]. The formula of percentage of incidence was calculated as follows, I (%) = (positive PCR plants/total of inoculated plants)*100. To determine the significant differences between groups we performed non-parametric Mann–Whitney test (P ≤ 0.05).

##### C. Viral Load

The quantification of the PepGMV load was determined by qPCR and it was carried out using PepGMVCPq5 and PepGMVCPq3 primers that amplify 104 bp of the AV1 gene [47]. The endogenous gene used was 18S rRNA (accession number: X67238) [49]. The reaction mix (10 µL) consisted of 5 µL of SsoFast^™^EvaGreen (BioRad), 0.1 µL of specific primers (250 µM) and 1 µL of the DNA sample and was conducted in 96-well optical plates in the CFX96 qPCR Detection System (BioRad). The cycling parameters were as follows: 1 cycle at 95 °C/30 s and 39 cycles each consisting of 95 °C/0.05 s and 60 °C/20 s. Following amplification, a melting curve analysis program was performed to verify the amplified products by their specific melting temperatures (Tm) from 65.0 °C to 95.0 °C at 0.5 °C/0.05 s increments. For each sample, three replicates were amplified in parallel. Two standard curves were prepared: the first from the target gene (plasmid containing the PepGMV AV1 partial gene) and the second from the internal report (plasmid containing the18S rRNA gene). The plasmid was serially diluted 10-fold, from 10 ng DNA/µL. Standard curves were obtained by linear regression analyses of the threshold cycle (Ct) value of each of the three standard-dilution replicates over the log starting quantity of DNA. Results were expressed as the log of the ratio of the quantity of virus DNA [32,50]. Data acquisition and analyses were performed using the BioRad CFX software (version 2.0). qPCR efficacy (E) was calculated by the formula: E = eln 10/−s–1, where a slope (s) of −3.322 (E = 2) represents 100% efficacy. Viral load comparisons were performed by one-way ANOVA analyses using SSPS statistical software (15.0).

#### 3.2.3. Determination of the Efficacy of RNAi Constructs

The efficacy (E) of the RNAi system was calculated as a percentage of reduction of the disease in protected plants with respect to unprotected plants and based on the ability to decrease the disease severity, incidence and viral load. The efficacy of variables was calculated with the formula: E (%) = (value of positive control for each variable – value of treatments (CIRT and CIRP) for each variable). 

### 3.3. Microarray Data

Per treatment, i.e. CIRT, CIRP and positive control, two independent biological replicates were used to monitor differences in gene expression between treatments. For each biological replicate, three leaves from each plant per treatment were harvested, pooled, flash-frozen, ground in liquid nitrogen, and stored at –80 ºC. Total RNA was extracted using TRIzol (Invitrogen). Of total RNA, 10 µg were used for cDNA synthesis with incorporation of fluorophores Alexa fluor 555 and Alexa fluor 647. An equal quantity of labeled cDNA from either CIRT or CIRP was hybridized, together with labeled cDNA from controls, to the collection of ‘*Arabidopsis thaliana* V.3.0.3. microarrays’ for 14 h at 42 ºC, using hybridization solution *UniHyb* (Arraylt). Data acquisition, processing and quantification of the images obtained from the microarray hybridization were performed in an Array-Pro Analizer 4000 with the corresponding *software* (Packard BioChips). To perform the analysis of microarray data and select genes that are significantly differentially expressed between classes of samples, we used the genArise package developed by the Computer Unit of the Institute of Cellular Physiology (UNAM). Data were normalized and the mean and standard deviation of the distribution of *log*_2_ (ratio) values were calculated, defining a global fold-change difference and confidence. Genes were considered to be differentially expressed with a q value ≤0.05 and a fold change cut off of 1.5 (Z-score). The databases Arabidopsis Information Resource (TAIR), Blast2GO, Database for Annotation Visualization and Integrate Discovery (DAVID) V6 were used to analyze Gene Ontology (GO). The microarrays data have been submitted to the Gene Expression Omnibus (http://www.ncbi.nlm.nih.gov/geo/).

## 4. Conclusions

This study shows that CIRP and CIRT constructs are efficient to induce resistance against infections caused by PepGMV in *N. benthamiana*. We found several up-regulated key components of the RNAi pathway. This demonstrates that the protection efficacy is due to the activation of the gene silencing mechanism, inducing efficiently an RNA-mediated cross-protection. Heterologous RNAi constructs are an interesting model to study antiviral strategies, a step forward for the control of PepGMV, and to understand the complex interaction between *Begomovirus*, RNAi, and the host.
